# Low Anterior Cervical Approach Without Sternotomy or Clavicle Resection for Upper Thoracic Vertebra Corpectomy

**DOI:** 10.7759/cureus.19329

**Published:** 2021-11-07

**Authors:** Denis Babici, Phillip M Johansen, Nikolas Echeverry, Koushik Mantripragada, Timothy Miller, Brian Snelling

**Affiliations:** 1 Neurology, Florida Atlantic University Charles E. Schmidt College of Medicine, Boca Raton, USA; 2 Neurological Surgery, Florida Atlantic University Charles E. Schmidt College of Medicine, Boca Raton, USA; 3 Neurological Surgery, Boca Raton Regional Hospital, Boca Raton, USA; 4 Neurosurgery, Boca Raton Regional Hospital, Boca Raton, USA

**Keywords:** invasive colon cancer, clinical skill under microscope, mri images, thoracic spine metastases. 8 gy in one fraction, vertebra, head neck cancer, anterior cervical corpectomy

## Abstract

The spine is the third most common site for metastatic disease following the lung and the liver. Approximately 60-70% of patients with metastatic cancer will have metastasis to the spine, but only 10% of these will be symptomatic. Metastases to the spine may involve the bone, epidural space, or the spinal cord. While chemotherapy and radiation therapy are the primary treatments for metastatic disease, spinal cord compression is an indication for surgical intervention. For vertebral body lesions, anterior vertebral reconstruction and stabilization also have the advantage of providing immediate stability to the vertebral column, but this anterior surgical approach to the upper thoracic spine is fraught with complications. The approach typically involves some combination of thoracotomy, sternotomy, or clavicle resection with anterior dissection into the superior mediastinum. To avoid unnecessary sternotomy and its associated complications, surgical access without sternotomy can be performed in certain cases. A sagittal MRI scan of the spine can be used to evaluate the level of the sternal notch in relation to the upper thoracic spine. If a tangential line can be drawn superior to the sternal notch and inferior to the level of the involved vertebra, surgical access without sternotomy can be performed. We present a case of a 52-year-old female with metastases to the upper thoracic vertebrae who underwent successful T2 corpectomy and T1-3 anterior fusion via a low anterior cervical approach, without sternotomy or clavicle resection.

## Introduction

The skeletal system is the third most common site of metastasis in the body, after the lung and liver. Within the skeletal system, the vertebral column is the most frequent site of metastasis [1]. Metastatic spinal cord compression often presents with symptoms of myelopathy or radiculopathy. The unique presentation is dependent on the exact location of the compression. Findings may include changes in fine motor skills such as handwriting, but problems with balance and gait can also be significant issues [2]. Upon initial presentation but before the diagnosis of metastatic bone disease is made, it is common for physicians to start patients on physical therapy and oral analgesics, under the assumption that the patient has cervical spondylosis or a disk herniation [1]. However, as was the case with the presented patient, these interventions fail to treat the underlying cause of symptoms so the patient will rarely see any improvement. Selecting the best treatment approach in these patients can be difficult, but appropriate management typically involves a multidisciplinary team consisting of an oncologist, radiation oncologist, and neurosurgeon or spine surgeon, along with the patient and their family. Multiple factors must be considered, but the patient’s prognosis and the stability of the spine should always be among the first [1,3]. Surgery may be considered, especially in cases of spinal cord compression. However, appropriate patient selection is necessary for improved surgical outcomes, characterized by restored spinal stability and improved quality of life [1].

Addressing metastatic lesions that involve the vertebral body in the upper thoracic spine is very challenging. During operative planning, the surgeon must consider many different surgical comorbidities, such as subclavian vessel damage, thoracic duct damage, and recurrent laryngeal nerve damage, which are associated with approaches that require splitting of the manubrium and clavicle [4]. An anterior, trans-clavicular, or trans-sternal approach may be considered, depending on the patient’s anatomy and the location of the lesion [5]. However, any approach that avoids sternotomy and clavicle resection is preferred to reduce the risks of surgical complications. By taking into consideration the spatial relationship of the affected vertebra with the manubrium, which can be done with a pre-operative sagittal MRI, the need for sternotomy can be avoided without compromising surgical decompression and reconstruction [6,7].

## Case presentation

**Figure 1:**
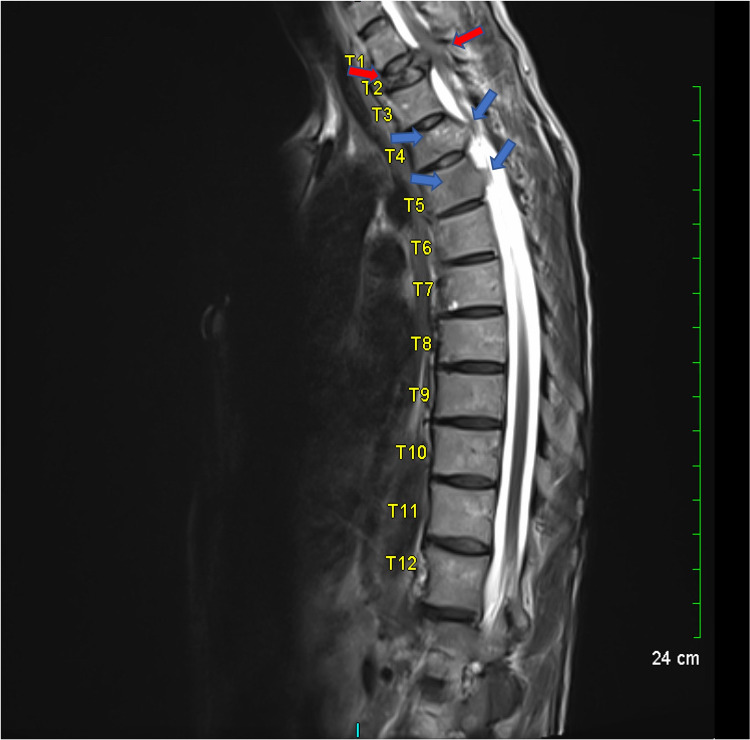
Sagittal MRI of the thoracic spine Severe compression fracture of the T2 vertebral associated with an expansile, epidural mass causing compression of the spinal cord (red arrows). An infiltrative process involving T4 and T5 vertebral bodies (blue arrows).

A 56-year-old female with no significant past medical history and no past surgical history was referred to the emergency department by an outpatient neurology clinic for concerns of transverse myelitis. The patient stated that two weeks prior, she woke up feeling bilateral lower extremity weakness associated with numbness and tingling extending from her feet up to her abdomen, in addition to abdominal dysesthesia. Prior to the onset of her neurological symptoms, the patient reported a history of intermittent back pain with no additional symptoms. The patient went to an urgent care facility where she was prescribed steroids, pain killers, and muscle relaxants without significant improvement. She left with instructions to follow-up with an orthopedic surgeon, who recommended her to follow-up with neurology due to her significant neurologic deficits. At the neurology clinic, strength was found to be four of five in her hip flexors bilaterally with associated numbness and tingling in her bilateral lower extremities. She was also found to have a sensory loss in the thorax centering around the T3 dermatome. The patient denied any bowel or bladder incontinence as well as any saddle anesthesia. Based on the physical examination findings, there was a concern for transverse myelitis, and the patient was sent to the emergency department for further investigation. On admission, the patient was hemodynamically stable and laboratory analysis was unremarkable. She noted mild back pain but denied blurry or double vision, loss of vision, or a history of optic neuritis. She also denied hiccups, nausea, vomiting, and a history of recent infection. Physical examination was notable for sensory loss at the approximate location of the T3 dermatome. Sagittal MRI of the thoracic spine showed a severe compression fracture of the T2 vertebral body with an associated expansile, epidural mass causing compression of the spinal cord, and infiltrative masses involving the T4 and T5 vertebral bodies (Figure 1). 

**Figure 2:**
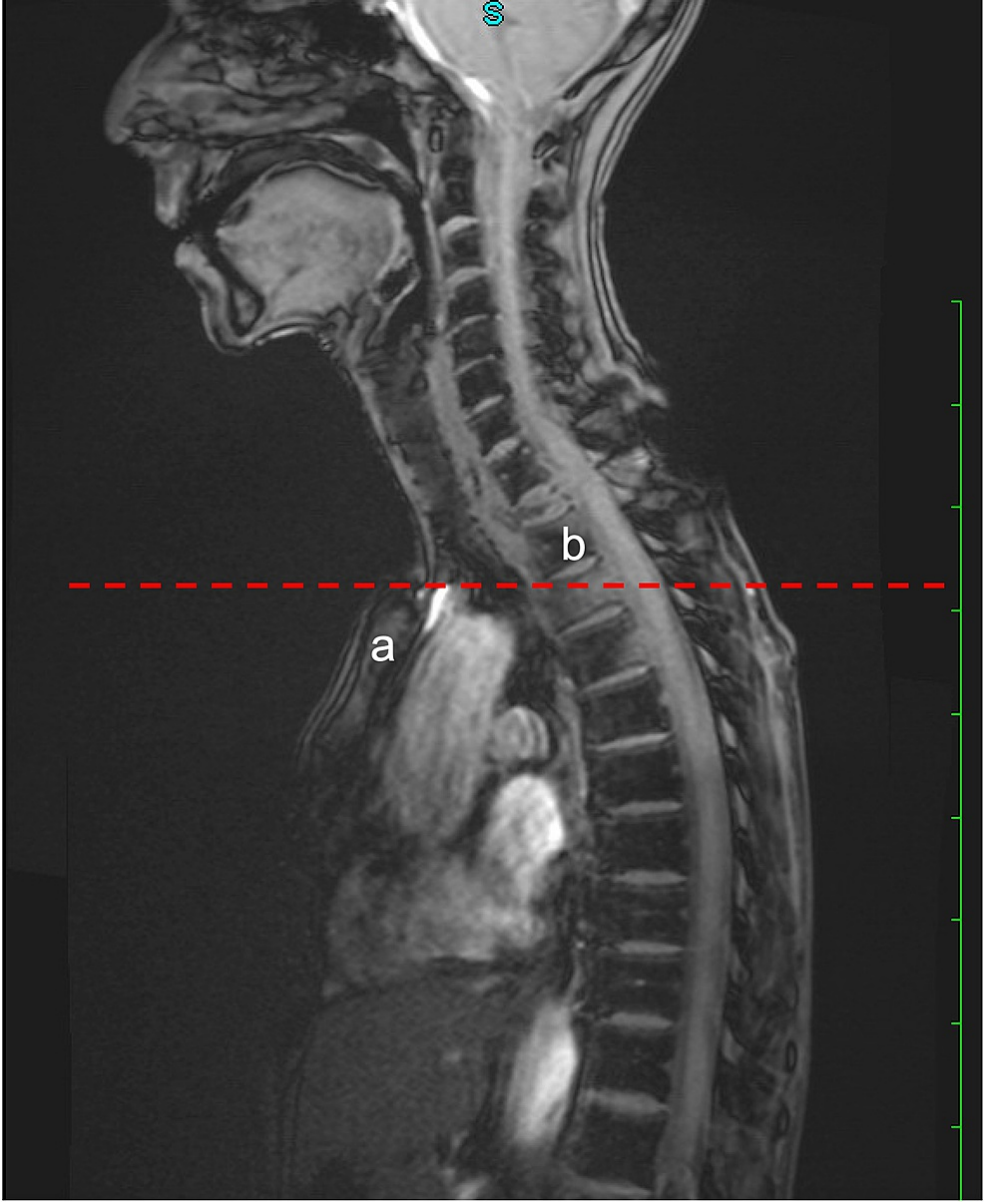
Sagittal MRI of the thoracic spine A tangential line is drawn superior to the sternal notch (a) and inferior to the level of the T3 vertebra (b).

**Figure 3:**
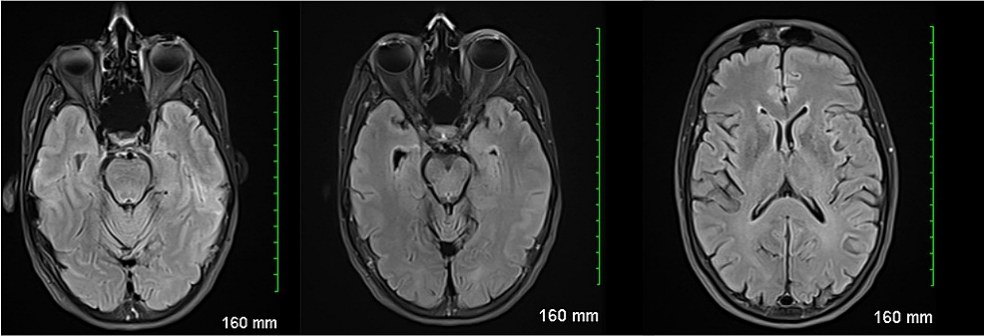
MRI of the brain is normal

A tangential line could be drawn superior to the sternal notch and inferior to the level of the T3 vertebra, confirming that access to the upper vertebrae was possible without sternotomy (Figure 2). MRI of the brain was unremarkable (Figure 3). 

**Figure 4:**
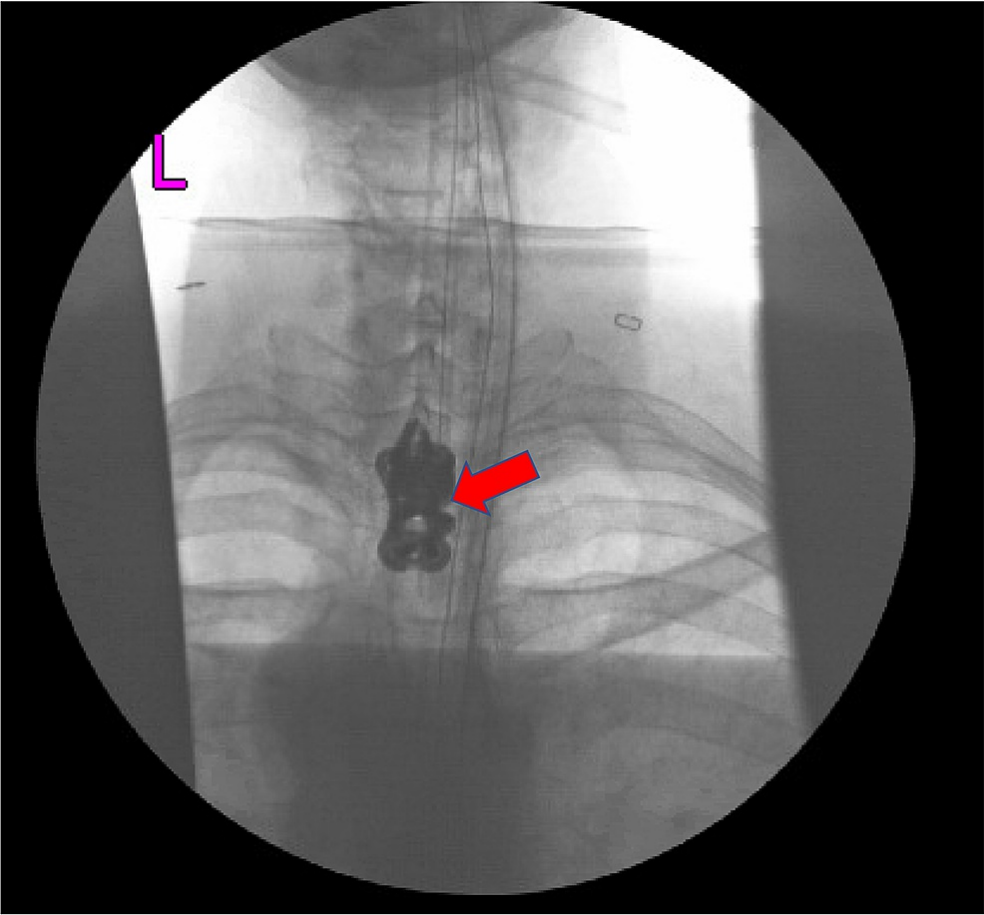
Anterior-posterior intraoperative fluoroscopy of the thoracic spine showing interbody cage between T1 and T3 (red arrow)

**Figure 5:**
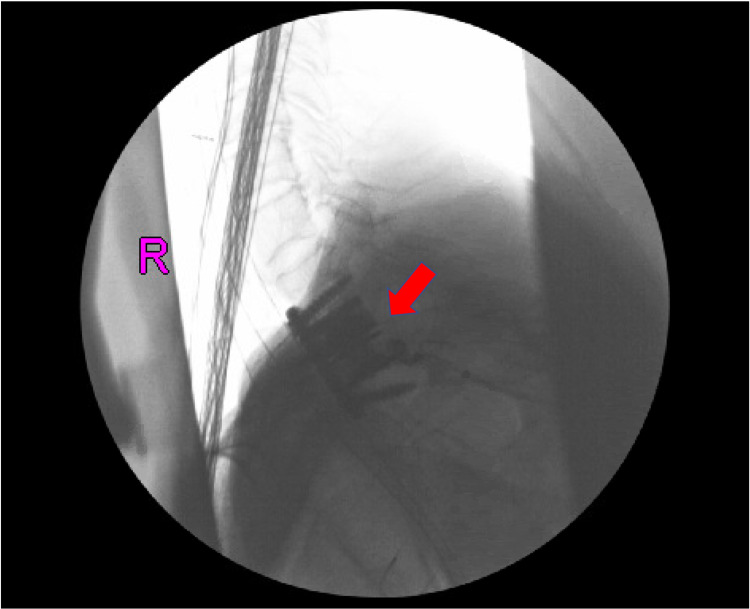
Lateral intraoperative fluoroscopy of the thoracic spine showing interbody cage between T1 and T3 (red arrow)

Based on the MRI findings and relation of the affected vertebra to the sternal notch, the decision was made to use an anterior approach to the T2 vertebral body via the low anterior cervical approach, without sternotomy or clavicle resection. A transverse neck incision was made medial to the left sternocleidomastoid muscle and superior to the left clavicle. The medial head of the sternocleidomastoid was mobilized from the clavicle. The sternohyoid and sternothyroid muscles were also identified and mobilized. Blunt dissection was performed medial to the carotid sheath, ensuring that the trachea and esophagus were retracted medially. The prevertebral fascia was carefully separated using blunt dissection until the anterior longitudinal ligament and the disc spaces were identified. The operative microscope was brought to the field, and the T2 vertebral body was noted to be almost completely collapsed secondary to tumor erosion. Discectomies were performed at the T1-T2 and T2-T3 levels by gently elevating the disc material away from the spinal cord. After complete T2 corpectomy and resection of the tumor, an interbody cage was placed between T1 and T3 to restore the height of the vertebral column. Proper positioning of the cage was confirmed using both anterior-posterior (Figure 4) and lateral intraoperative fluoroscopy (Figure 5). 

After placement of the interbody cage, a separate anterior plate spanning the level of T1-T3 was placed, and titanium screws were used to secure the plate to the T1 and T3 vertebral bodies. The focus was then shifted to the T4 and T5 lesions, which were addressed utilizing a posterior approach. The left T4 pedicle, which appeared to be engulfed by a tumor, was removed. A T5 laminectomy was also completed to decompress the dorsal and lateral spinal cord. The dura ventrally, laterally, and dorsally was inspected and found to be free of any further tumor compression. Pedicle screw instrumentation was placed and affixed to rods using stereotactic navigation from T1 to T6, skipping T2 bilaterally and T4 on the left.

**Figure 6:**
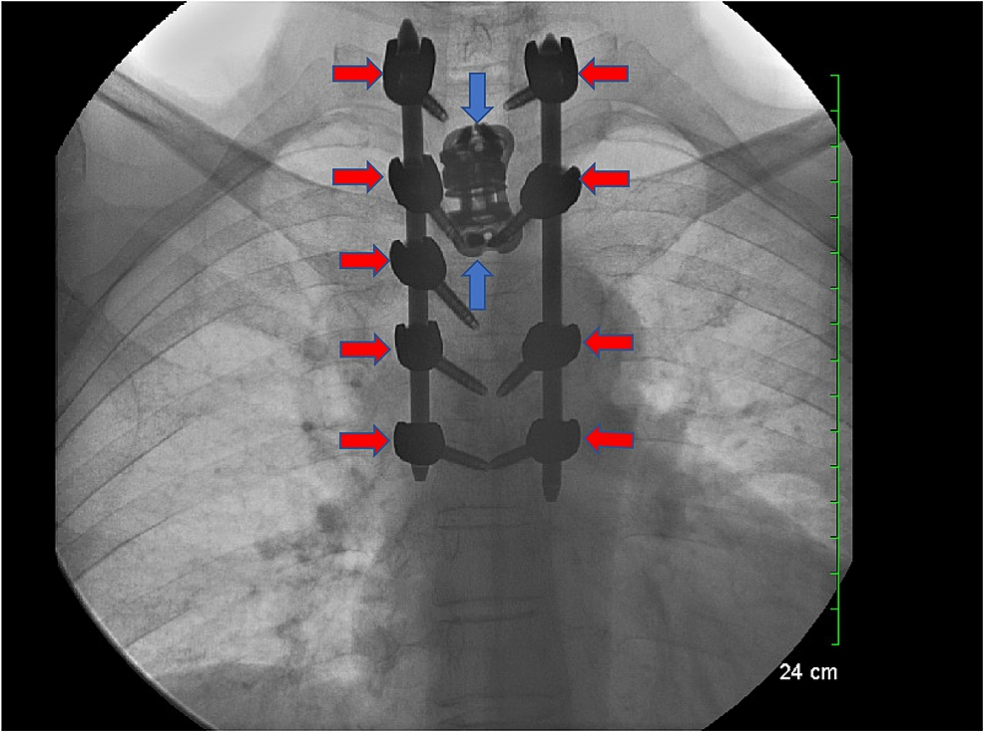
Postoperative anterior-posterior XR of the thoracic spine showing proper positioning of the wires (red arrows) and interbody cage (blue arrows) XR: x-ray

The postoperative period proceeded without complications. The permanent specimen was found to be metastatic adenocarcinoma. The patient was discharged home on postoperative day five with outpatient oncology follow-up to determine the primary source of the metastasis and plan for treatment. After two weeks, the patient returned for outpatient follow-up. X-ray (XR) showed intact hardware in good positioning (Figure 6).

## Discussion

Surgical approaches to vertebral body lesions around the upper thoracic spine are technically challenging procedures because of the vertebral bodies’ relative inaccessibility. Different approaches have been proposed to provide safe and effective access to this region [8-12], but the most common approach to upper thoracic corpectomy is the anterior approach, which leaves the vertebrae buried behind the numerous anterior anatomic structures [13]. This patient’s sagittal MRI of the thoracic spine showed a severe compression fracture of the T2 vertebral body with an associated expansile, epidural mass causing compression of the spinal cord. To ensure adequate surgical access to the upper thoracic vertebrae, previous studies suggest using sagittal MRI of the spine to evaluate the level of the sternal notch in relation to the upper thoracic spine [6,7,14,15]. If a tangential line can be drawn superior to the sternal notch and inferior to the level of the involved vertebra, access to the upper thoracic vertebra can be obtained without sternotomy. Such was the case in this patient, indicating that her surgery could be performed using a left-sided, low anterior cervical approach without sternotomy or clavicle resection. This approach can be utilized based on analysis of pre-operative imaging, like in the case of this patient. When feasible, the low anterior approach provides similar clinical outcomes with lower rates of surgical morbidity compared to methods that involve sternotomy or clavicle resection [8,12]. In addition, this less invasive approach is familiar to most spinal surgeons and still provides a suitable working field while simultaneously avoiding unnecessary dissection of nearby vascular and neural structures.

A low anterior approach to the upper thoracic spine reduces the risk of surgical complications associated with more invasive trans-sternal or trans-clavicular approaches. Vocal cord paresis, secondary to injury of the recurrent laryngeal nerve, is one of the more serious surgical complications [16]. The reported incidence of recurrent laryngeal nerve damage ranges from 4.76% to 16.67% [17] and is due to factors like direct damage during sharp dissection or overstretching of the nerves, especially at the recurrent point of the recurrent laryngeal nerve [13]. The left-sided anterior approach to the upper thoracic region is preferable to decrease the risk of iatrogenic recurrent laryngeal nerve injury because the left recurrent laryngeal nerve has a longer route and has relatively fixed anatomy in the tracheoesophageal groove. In contrast, the right recurrent laryngeal nerve has substantial variations and is more likely to be unrestrained within the mediastinum [18].

## Conclusions

In patients with upper thoracic vertebral body lesions who require surgical decompression and stabilization, pre-operative sagittal MRI scan of the thoracic spine can affirm the possibility of a low anterior cervical approach without sternotomy, thereby decreasing complications related to more invasive approaches. This low anterior approach helps avoid injuries to subclavian vessels and the thoracic duct, which are usually secondary to resection of the clavicle or disruption of the sternoclavicular junction, and it also decreases the likelihood of iatrogenic injury to the recurrent laryngeal nerve.
